# Influence of the Expression Level of O^6^-Alkylguanine-DNA Alkyltransferase on the Formation of DNA Interstrand Crosslinks Induced by Chloroethylnitrosoureas in Cells: A Quantitation Using High-Performance Liquid Chromatography-Mass Spectrometry

**DOI:** 10.1371/journal.pone.0121225

**Published:** 2015-03-23

**Authors:** Lili Li, Sisi Li, Guohui Sun, Ruizeng Peng, Lijiao Zhao, Rugang Zhong

**Affiliations:** Beijing Key Laboratory of Environmental and Viral Oncology, College of Life Science and Bioengineering, Beijing University of Technology, Beijing, P. R. China; Institute of Molecular Genetics IMG-CNR, ITALY

## Abstract

Chloroethylnitrosoureas (CENUs), which are bifunctional alkylating agents widely used in the clinical treatment of cancer, exert anticancer activity by inducing crosslink within a guanine-cytosine DNA base pair. However, the formation of dG-dC crosslinks can be prevented by O^6^-alkylguanine-DNA alkyltransferase (AGT), ultimately leading to drug resistance. Therefore, the level of AGT expression is related to the formation of dG-dC crosslinks and the sensitivity of cells to CENUs. In this work, we determined the CENU-induced dG-dC crosslink in mouse L1210 leukemia cells and in human glioblastoma cells (SF-763, SF-767 and SF-126) containing different levels of AGT using high-performance liquid chromatography coupled with electrospray ionization tandem mass spectrometry. The results indicate that nimustine (ACNU) induced more dG-dC crosslinks in L1210 leukemia cells than those induced by carmustine (BCNU), lomustine (CCNU) and fotemustine (FTMS). This result was consistent with a previously reported cohort study, which demonstrated that ACNU had a better survival gain than BCNU, CCNU and FTMS for patients with high-grade glioma. Moreover, we compared the crosslinking levels and the cytotoxicity in SF-763, SF-767 and SF-126 cells with different AGT expression levels after exposure to ACNU. The levels of dG-dC crosslink in SF-126 cells (low AGT expression) were significantly higher than those in SF-767 (medium AGT expression) and SF-763 (high AGT expression) cells at each time point. Correspondingly, the cytotoxicity of SF-126 was the highest followed by SF-767 and SF-763. The results obtained in this work provided unequivocal evidence for drug resistance to CENUs induced by AGT-mediated repair of DNA ICLs. We postulate that the level of dG-dC crosslink has the potential to be employed as a biomarker for estimating drug resistance and anticancer efficiencies of novel CENU chemotherapies.

## Introduction

**Fig 1:**
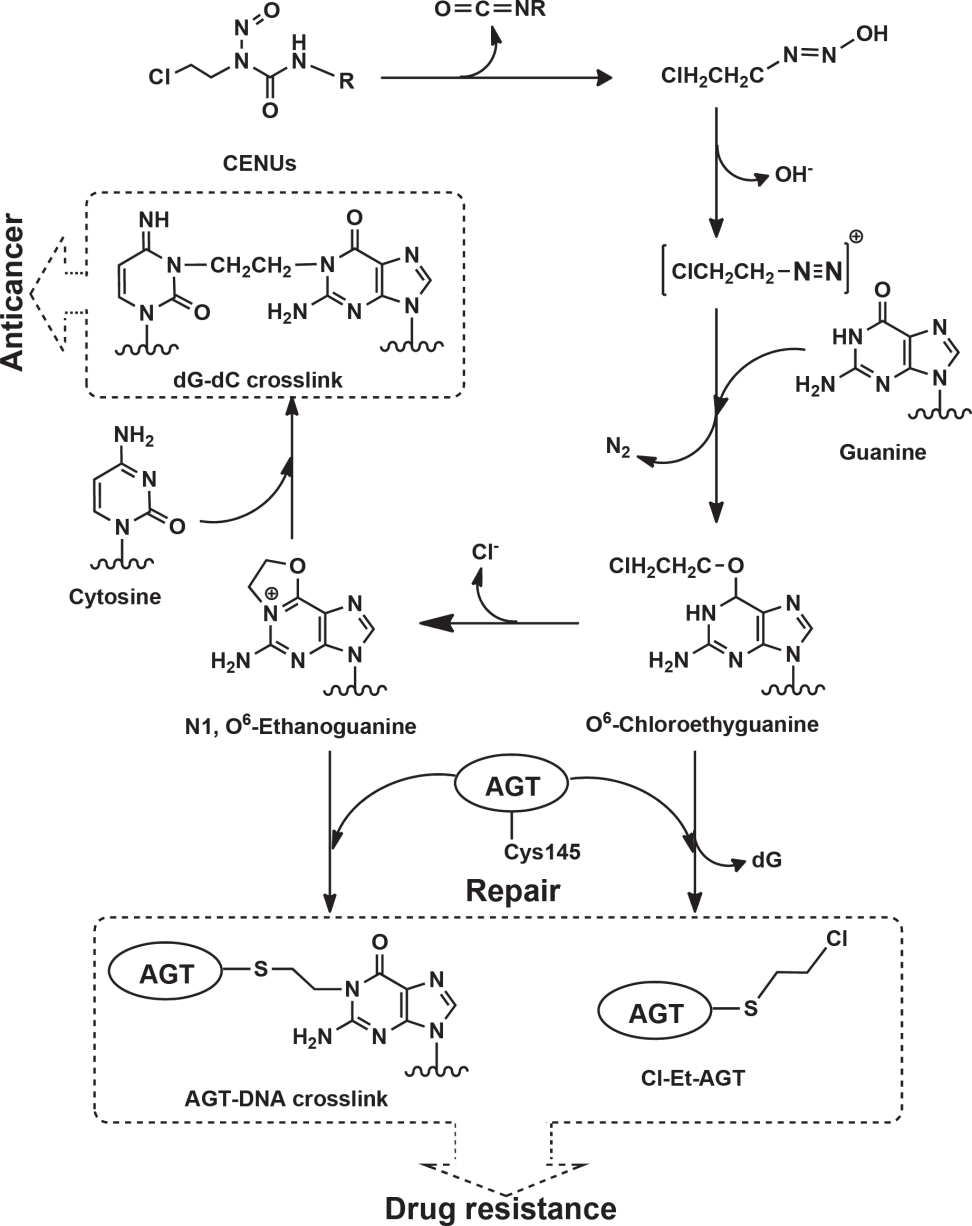
Supposed mechanisms for the formation of dG-dC crosslinks induced by CENUs and the repair of crosslinks mediated by AGT.

Chloroethylnitrosoureas (CENUs) are bifunctional anti-tumor alkylating agents that have important clinical applications for the treatment of cancer, such as lymphomas, melanomas, and cerebromas [1–3]. Typical CENU chemotherapies used in clinical applications include carmustine (BCNU), lomustine (CCNU), semustine (MeCCNU), nimustine (ACNU) and fotemustine (FTMS) (see S1 Table). CENUs have high lipophilicity and can cross the blood-brain barrier, thus they are frequently used as chemotheraputics for brain tumors [4,5]. *In vivo* evidence indicated that CENUs possessed high activity against intracerebrally inoculated L1210 leukemia and prolonged the survival of mice [6–8]. CENUs are unstable under physiological conditions and spontaneously undergo decomposition to yield active chloroethylating species [9–10]. These active electrophilic agents are capable of alkylating DNA and, further, leading to interstrand crosslinks (ICLs) [11–14]. Fig. 1 shows the supposed mechanism for the formation of ICLs induced by CENUs, in which guanine was alkylated by the chloroethyldiazonium ion to form O^6^-(2-chloroethyl)-deoxyguanosine (O^6^-ClEtdGuo) followed by the second alkylation of the complementary deoxycytidine to form dG-dC crosslinks via the intermediate N1,O^6^-ethano-deoxyguanosine (N1,O^6^-EtdGuo) [15,16]. The dG-dC crosslinks are believed to be the most cytotoxic lesions and responsible for the antitumor activities of CENUs because the crosslinks inhibit strands separation during DNA replication and transcription, leading to apoptosis if not repaired correctly. It was estimated that a single ICL could kill a repair-deficient bacterial or yeast cell, and as few as 20 to 40 ICLs can be lethal to a mammalian cell lacking the ability to remove the crosslinks [17–19].

CENU-induced dG-dC crosslinks are poorly repaired once formed. However, O^6^-alkylguanine DNA alkyltransferase (AGT) provides a unique means for DNA repair by directly transferring the chloroethyl group located at the O^6^ position of guanine to the cysteine145 residue of AGT before the formation of a crosslink (see Fig. 1) [20–23]. *In vitro* and *in vivo* evidence has demonstrated that a high expression level of AGT in tumor cells was the primary reason leading to resistance to CENUs, and increased levels of AGT appeared to correlate well with the elevated resistance of tumor cells to chloroethylating agents [24–27]. Penketh et al. [28] investigated the dG-dC crosslinks induced by Cloretazine, which is a short-lived prodrug (t_1/2_∼30 s at pH 7.4 and 37°C) with a similar anticancer mechanism to CENUs. The results indicated that Cloretazine induced double the amounts of dG-dC crosslinks in AGT-deficient L1210 and U937 cells than in AGT-proficient HL-60 cells. Bodell et al. [29,30] also found that the levels of dG-dC crosslink formed in AGT-proficient 9L-2, HU-188, and HU-252–2 rat glial cells were approximately 50% of those in AGT-deficient 9L and HU-126 cells. This evidence suggests that AGT repair is a major factor related to the antineoplastic efficacy of CENUs.

To assess the toxicity, mutagenicity, and pharmacological response of various CENUs and obtain a better understanding of the relationship between the chemotherapeutic effects of CENUs and the repair capacity of cellular DNA, sensitive methods are required to identify and quantify dG-dC crosslinks. However, clinical studies are often hampered by the absence of a sensitive method to detect this important DNA lesion. High-performance liquid chromatography coupled with tandem mass spectrometry (HPLC-MS/MS) is considered to be a robust technique with higher sensitivity and specificity compared with chromatographic, electrophoretic and fluorescent assays in the quantification of DNA adducts. In our previous studies, HPLC-MS/MS was employed to determine the yield of dG-dC crosslinks induced by MeCCNU in calf thymus DNA [31], and induced by ACNU in NIH/3T3 and L1210 cells [32]. In this work, we reported an HPLC-MS/MS quantification of dG-dC crosslink induced by ACNU, BCNU, CCNU and FTMS in SF-763, SF-767, SF-126 and L1210 cells containing different levels of AGT. This work is expected to provide a robust method for determining ICLs in cells and clinical biospecimens and assist in evaluating the cellular resistance to novel CENU chemotherapies.

## Experimental

### Chemicals and Materials

ACNU, BCNU, CCNU, FTMS, acetonitrile (HPLC grade), 2'-deoxyguanosine, 2'-deoxycytidine and phosphodiesterase I were obtained from Sigma-Aldrich (St. Louis, MO, USA). Nuclease S1, alkaline phosphatase (CIAP) and deoxyribonuclease I were purchased from TaKaRa Biotechnology (Tokyo, Japan). ^15^N_3_-2'-deoxycytidine was acquired from Cambridge Isotope Laboratories (Andover, MA, USA). The dG-dC standard and isotope-labeled ^15^N_3_-dG-dC internal standard were prepared as previously described with minor modifications [33]. All other chemicals, reagents and solvents were obtained from Sigma-Aldrich. Microcon YM-10 centrifuge columns were purchased from Millipore (Billerica, MA, USA). Deionized water was purified using a PALL deionizer.

Murine L1210 lymphoid leukemia cells and human SF-126, SF-763 and SF-767 glioma cells were obtained from the Cell Center of Peking Union Medical College (Beijing, China). All cell culture media and reagents were purchased from HyClone Laboratories (Logan, UT, USA).

### Cell culture

All cells were maintained and treated at 37°C in a humidified 5% CO_2_ and 95% air atmosphere. Mouse L1210 leukemia cells were grown in high glucose Dulbecco's modified Eagle's medium (DMEM) supplemented with 10% (v/v) horse serum (HS) and antibiotics (100 U/mL penicillin and 100 μg/mL streptomycin). Human SF-126, SF-763 and SF-767 glioma cells were cultured in high glucose Eagle’s minimum essential medium (MEM) supplemented with 10% (v/v) fetal calf serum (FCS) and antibiotics (100 U/mL penicillin and 100 μg/mL streptomycin). Cells were passaged as required every 2 to 3 days. The culture medium was discarded when cells achieved a saturation density of 1∼2×10^6^ cells/mL. The cells were washed twice with D-Hank’s basal salt solution, and then fresh medium containing the drugs was added.

### Treatment of L1210 cells with ACNU, BCNU, CCNU and FTMS

A stock solution of ACNU was freshly prepared in deionized water and diluted with DMEM to obtain final concentrations of 0.025, 0.05, 0.1, 0.2 and 0.4 mM. L1210 cells were incubated in culture solutions containing ACNU at 37°C for various treatment times (3, 6, 9 and 12 h). For the control groups, cells were incubated under the same conditions except without the addition of ACNU. Cells were collected by centrifugation at each time point and washed twice with phosphate-buffered saline (PBS). The obtained cellular pellets were kept at-20°C until DNA extraction. The treatments of L1210 cells with BCNU, CCNU and FTMS were carried out using the same protocol as ACNU, except the drugs were dissolved in ethanol.

### Treatment of SF-126, SF-763 and SF-767 cells with ACNU

ACNU was dissolved in deionized water immediately prior to use and directly added into fresh medium to achieve the final concentrations of 0.2, 0.6 and 1 mM. Cells were grown in the culture solutions containing ACNU at 37°C for various treatment times (6, 12, 18, and 24 h). For each time point, the control cells were incubated under the same conditions for the same period of time but without ACNU. Cells were detached using trypsin-EDTA and harvested by centrifugation. Next, the obtained cellular pellets were washed with PBS and stored at-20°C until DNA isolation.

### Cytotoxicity assay

Cytotoxicity was determined using the trypan blue exclusion assay. The dead cells were stained blue and were easily distinguished from the viable cells excluding the dye. The cell viability rate was determined using a Cellometer automatic cell counter (Nexcelom Bioscience LLC, Lawrence, MA). The cell death rate was calculated according to formula (1), in which V refers to the cell viability of the treated groups, and V_0_ is the cell viability of the control group.

$$\text{Cell death rate (\%) = (1 - }\frac{\text{V}}{{\text{ V}}_{\text{0}}})\times 100$$ (1)

### DNA extraction and enzymatic digestion

DNA extraction was performed as described in previous studies [34,35], with some modification. Briefly, cells were homogenized in 10 mL of lysis buffer, which contained 10 mM Tris-HCl, 0.1 M EDTA and 0.5% (w/v) sodium dodecyl sulfate (SDS). To cell lysates was then added 60 μL of proteinase K (20 mg/mL), which were then shaken at 37°C for 12 h. The mixture was extracted with phenol-chloroform-isoamyl alcohol solution (25:24:1, v/v/v) twice. Then, genomic DNA was precipitated from the cell lysate by adding 100% cold ethanol. All DNA samples were dried with a stream of nitrogen and stored at-20°C until enzymatic hydrolysis.

The isolated DNA was redissolved in 100 μL of Tris-HCl buffer (10 mM, pH 7.4). The purity of DNA was confirmed by measuring UV absorption at 230, 260 and 280 with the ratios of 260/230 and 260/280 at 2.4 and 1.8, respectively. Following the previously reported protocols [32], DNA samples were digested with four enzymes, including DNase I, nuclease S1, alkaline phosphatase and snake venom phosphodiesterase. Briefly, the solutions were heated at 98°C for 5 min and promptly chilled in an ice-bath for 10 min. Each solution (100 μL, containing 0.1 to 1 mg DNA) was hydrolyzed using 45 units of DNase I (15 μL, buffered in 20 mM CH_3_COONa, 150 mM NaCl, pH 5.0) and 100 units of nuclease S1 (10 μL, buffered in 10 mM CH_3_COONa, 150 mM NaCl, 0.05 mM ZnSO_4_, pH 4.6). After incubation at 37°C for 6 h, the mixture was further incubated overnight at 37°C with the addition of 20 units (20 μL) of alkaline phosphatase and 0.005 units of phosphodiesterase I (5 μL) buffered in 500 mM Tris-HCl, 10 mM MgCl_2_ (pH 9.0). Finally, the digestion mixtures were passed through a molecular weight centrifugal filter (Microcon YM-10) for HPLC-ESI-MS/MS analysis. A buffer control without DNA was prepared for each set of samples and processed as the negative controls following the same procedure.

### Quantification of dG-dC crosslinks in cells by HPLC-ESI-MS/MS

Quantification of dG-dC crosslinks in the DNA digestion mixture was performed by a Thermo TSQ Quantum Discovery MAX triple quadrupole tandem mass spectrometer interfaced with a Thermo Finnigan HPLC system (Thermo Finnigan, San Jose, CA). The dG-dC crosslinks were separated using a Zorbax SB-C18 column (2.1×150 mm, 5 μm particle size; Agilent Technologies, Palo Alto, CA) and eluted with a mobile phase consisting of deionized water containing 0.01% acetic acid (solvent A) and acetonitrile (solvent B) at a flow rate of 0.1 mL/min. The solvent gradient started from 2% B for the first 5 min, then was increased linearly to 80% B over 20 min and held for 3 min. Subsequently, the solvent composition was returned to 2% B over 2 min followed by a column equilibration for 15 min. The mass spectrometer was operated in positive mode with nitrogen as the sheath gas (50 psi) and argon as the collision gas (1 mTorr). The mass spectrometer was optimized to maximal sensitivity by infusing the ^15^N_3_-dG-dC internal standard. Typical instrument settings included a spray voltage of 4.0 kV, capillary temperature of 300°C, source CID of 8 V and collision energy of 20 V. The signals from dG-dC crosslinks and the ^15^N_3_-dG-dC internal standard were monitored by selecting reaction monitoring (SRM) with the transitions of m/z 521→289 and 524→292 for dG-dC and ^15^N_3_-dG-dC, respectively.

Quantification of dG-dC crosslinks in cells was performed using stable isotope dilution HPLC-ESI-MS/MS. The calibration curve was constructed by analyzing the standard solutions containing a fixed amount of ^15^N_3_-dG-dC (9.6 nM) mixed with increasing amounts of dG-dC (0.04, 0.1, 1, 8, 16, 32 and 50 nM). Solvent blanks were periodically injected to detect potential analyte carry-over. The calibration curve was obtained by plotting the SRM peak area ratios of dG-dC to ^15^N_3_-dG-dC versus the corresponding concentration ratios. Cells without CENU treatment were prepared for each group and processed as negative controls following the same procedure. The levels of dG-dC crosslink in the cell samples were expressed as fmol dG-dC crosslinks/mg DNA.

### Quantification of dG

The concentration of DNA was quantified by HPLC-UV analysis of dG in the enzymatic hydrolysate of DNA. The amount of DNA was calculated from the dG content by assuming that 1 mg of DNA contained 3 μmol of nucleotides and that dG accounted for 22% of the total nucleotides in DNA [36]. The experiment was conducted on a Thermo Finnigan HPLC system with a diode array detector. Gradient elution was performed using a Luna C18 column (4.6×250 mm, 5 μm in particle size, Phenomenex, Torrance, CA) eluted with deionized water (solvent A) and methanol (solvent B) at a flow rate of 0.7 mL/min. The UV signal was monitored at 254 nm. The solvent composition began at 5% B followed by linear increase to 22% B over 15 min. Then the solvent B was raised to 80% over 2 min followed by an isocratic elution for 3 min. Subsequently, the percentage of the solvent B was brought back to 5% over 2 min, followed by an equilibration for 15 min.

### Statistical Analysis

All statistical analysis was performed using the Microsoft Excel statistical software (Microsoft Office Excel 2010, Microsoft Corp., Redmond, WA). Comparisons of the levels of dG-dC crosslink were performed using t-tests between SF-126, SF-767 and SF-763 cells. A Pearson correlation analysis was performed between the levels of dG-dC crosslink using SPSS (Statistical Package for the Social Sciences) software. The levels of dG-dC crosslink were expressed as the mean values ± standard deviation (SD) of single analyses of three DNA samples per group. The cell death rates were also presented as the mean values ± SD of three biologically independent experiments for each group. Corrected P<0.05 and P<0.01 was interpreted to indicate significant and very significant differences, respectively, of the mean value comparison. Alpha was set to 0.05 to determine the statistical significance.

## Results and Discussion

### Method validation

**Fig 2:**
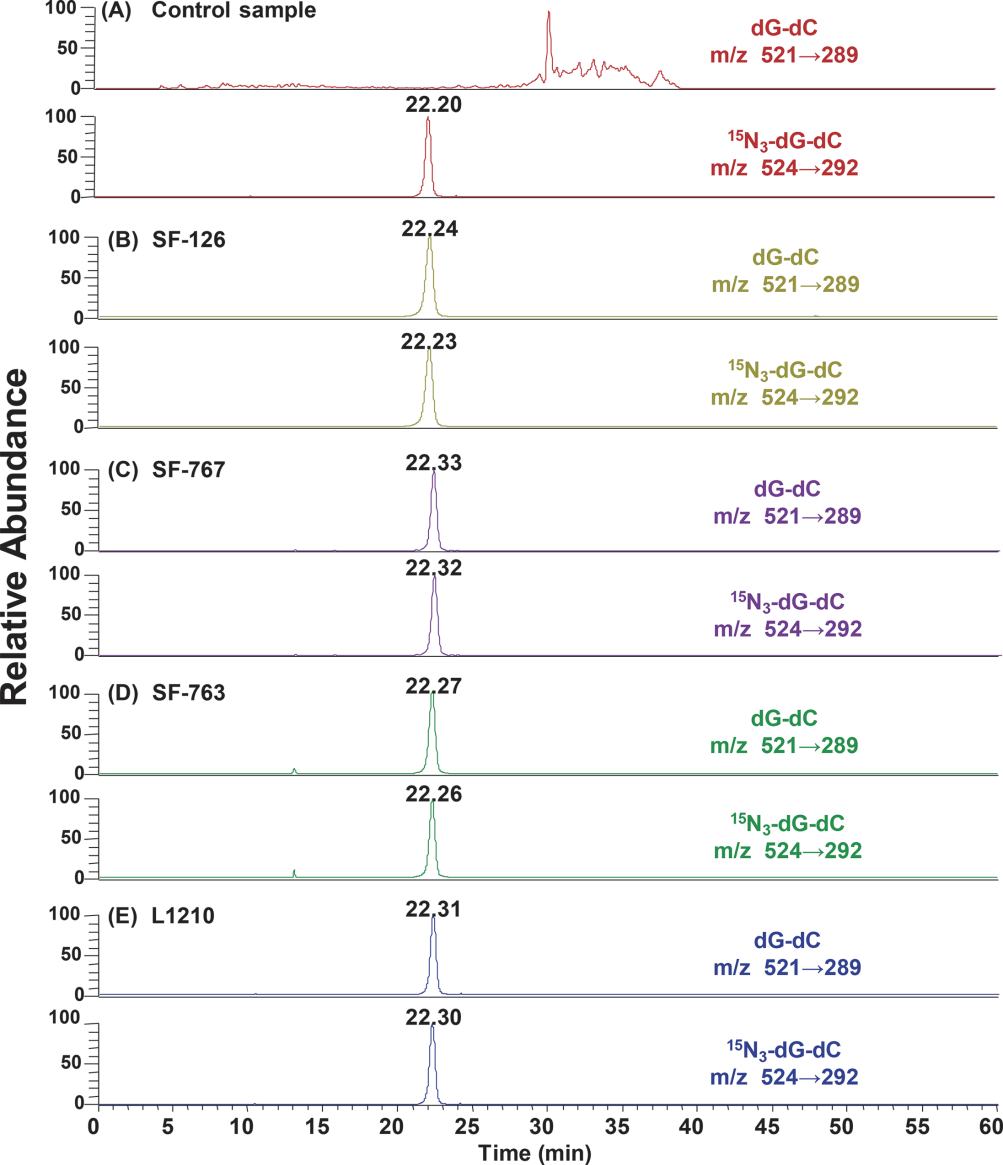
SRM chromatograms of dG-dC crosslinks in the DNA digestion mixtures from control cells (A) and ACNU-treated SF-126 (B), SF-767 (C), SF-763 (D) and L1210 (E) cells.

In our previous study [32], the HPLC-ESI-MS/MS method was successfully employed in the determination of dG-dC crosslinks in mouse leukemia L1210 cells and fibroblast NIH/3T3 cells treated with ACNU. In the present study, the method was used for quantifying the dG-dC crosslinks in human glioma SF-126, SF-763 and SF-767 cells treated with ACNU and in L1210 cells treated with four types of CENUs. The positive ESI product ion spectra (see S1 Fig.) indicated that m/z 289 and 292 were the main fragments of dG-dC (m/z 521) and ^15^N_3_-dG-dC (m/z 524), respectively. As shown in S2 Fig., the fractions of dG-dC standard and ^15^N_3_-dG-dC internal standard in the control DNA digestion mixture coeluted at 22 min with the transitions of m/z 521→289 and m/z 524→292, respectively. The limits of quantitation (LOD) and limits of detection (LOQ) were 2 and 8 fmol, defined as the amounts of dG-dC crosslink giving rise to signal-to-noise ratios of 5 and 15, respectively. As shown in S3 Fig., the calibration curve was linear over the range from 0.04 to 50 nM, with a correlation coefficient (R^2^) of 0.9999. Fig. 2B-E displays typical SRM chromatograms for the DNA enzymatic hydrolysates from SF-126, SF-767, SF-763 and L1210 cells treated with 0.6 mM ACNU for 12 h. The dG-dC and ^15^N_3_-dG-dC peaks in the digestion mixture coeluted at 22 min. In Fig. 2A, there was no signal for dG-dC crosslinks observed in the DNA hydrolysates from the control cells, which indicated that there was no significant matrix interference or contamination in the analyte channels from enzymatic hydrolysates or the internal standard. The above results demonstrated that the method employed in this work was robust for determining the CENU-induced dG-dC crosslinks in various cells, due to its high sensitivity and specificity.

### Quantitation of dG-dC crosslinks in L1210 cells treated with four CENUs

**Fig 3:**
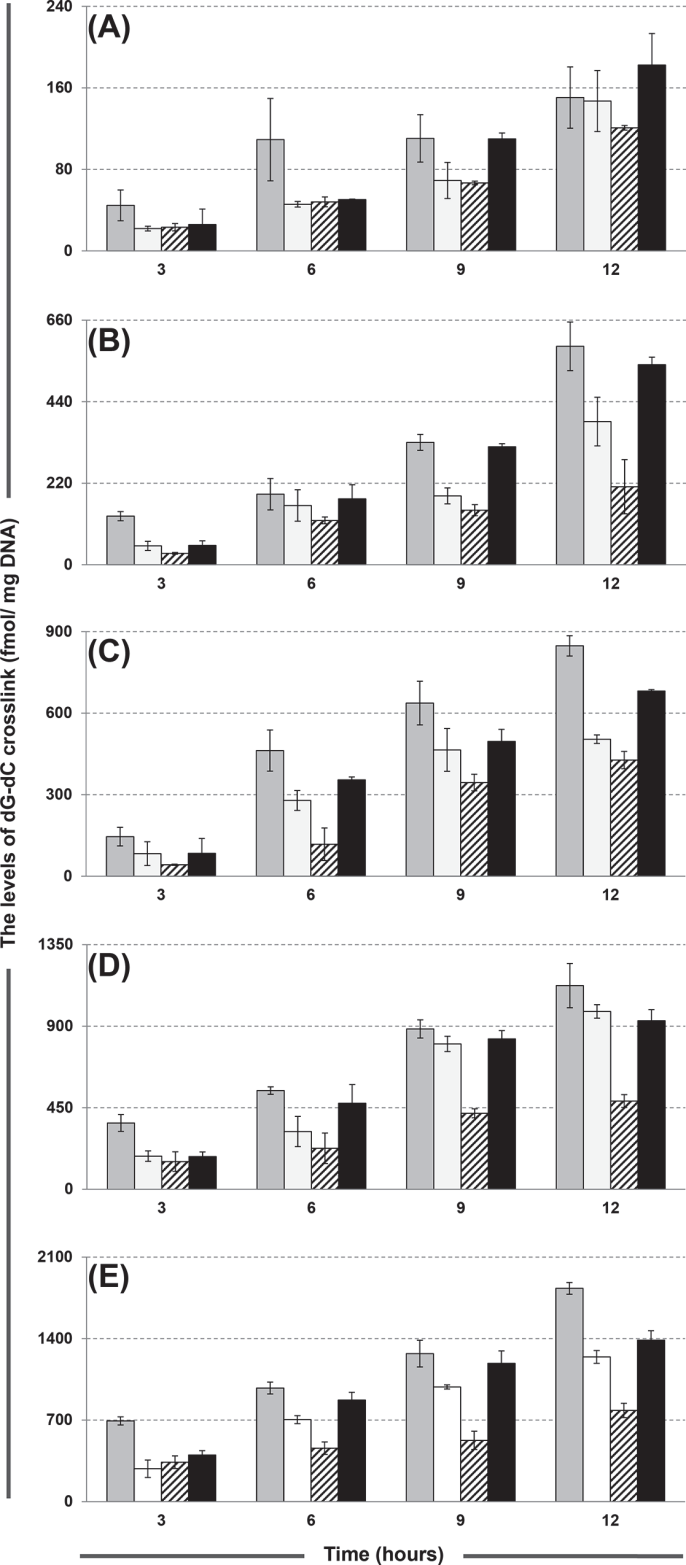
Comparison of the levels of dG-dC crosslink (fmol/mg DNA) induced by ACNU, BCNU, CCNU and FTMS in L1210 cells, represented by gray bars, open bars, striped bars and solid bars, respectively. The concentrations of the drugs are 0.025 (A), 0.05 (B), 0.1 (C), 0.2 (D) and 0.4 mM (E).

Fig. 3 shows the comparison of the dG-dC crosslinking levels in L1210 cells induced by the four CENUs at various concentrations. The corresponding values for the determined levels of dG-dC crosslink at each time point are listed in S2 Table. For the four CENUs, the levels of dG-dC crosslink all display dose-dependence with the drug concentration increased from 0.025 to 0.4 mM. The crosslinking levels exhibit a common increasing trend over 0 to 12 hours, without any sign of decrease. For ACNU, the maximal crosslinking levels at 12 h were 150, 589, 848, 1124 and 1832 fmol/mg DNA for the 0.025, 0.05, 0.1, 0.2 and 0.4 mM concentrations, respectively. For the groups treated with BCNU, CCNU and FTMS, the maximal crosslinking levels were 1243, 783 and 1387 fmol/mg DNA, respectively, which were observed at 12 h with the highest drug concentration.

Fig. 3 indicated that the amounts of dG-dC crosslink in L1210 cells treated with ACNU were higher than those treated with FTMS, BCNU and CCNU at all time points for all concentration groups, with the exception that FTMS induced a higher level than ACNU at 12 h for the 0.025 mM group (Fig. 3A). This result is consistent with the results of the previous investigation reported by Bodell et al. [37,38]. They determined the DNA ICLs in human glial-derived cells and 9L rat gliosarcoma cells treated with ACNU and BCNU using an alkaline elution assay, and observed that the crosslinking levels induced by ACNU were higher than those induced by BCNU in all cell lines. Because the cytotoxicity of CENUs is considered to be related to the formation of DNA ICLs, it was postulated that the anticancer activity of CENUs might be associated with the levels of dG-dC crosslink. Wolff et al. [39] compared the anticancer efficiency of ACNU, BCNU, CCNU, FTMS and other CENUs by performing a survival gain analysis of 364 studies describing 24,193 patients with high-grade glioma treated in 504 cohorts. They demonstrated that the highest survival gain (8.9 months) was achieved with ACNU treatment. Preuss et al. [40] demonstrated that ACNU was more selective than CCNU and BCNU in killing AGT-deficient tumor cells. The above investigations suggest that the levels of dG-dC crosslink might be a potent predictor for estimating the anticancer activity of CENUs.

### Quantitation of dG-dC crosslinks in SF-767, SF-763 and SF-126 cells treated with ACNU

**Fig 4:**
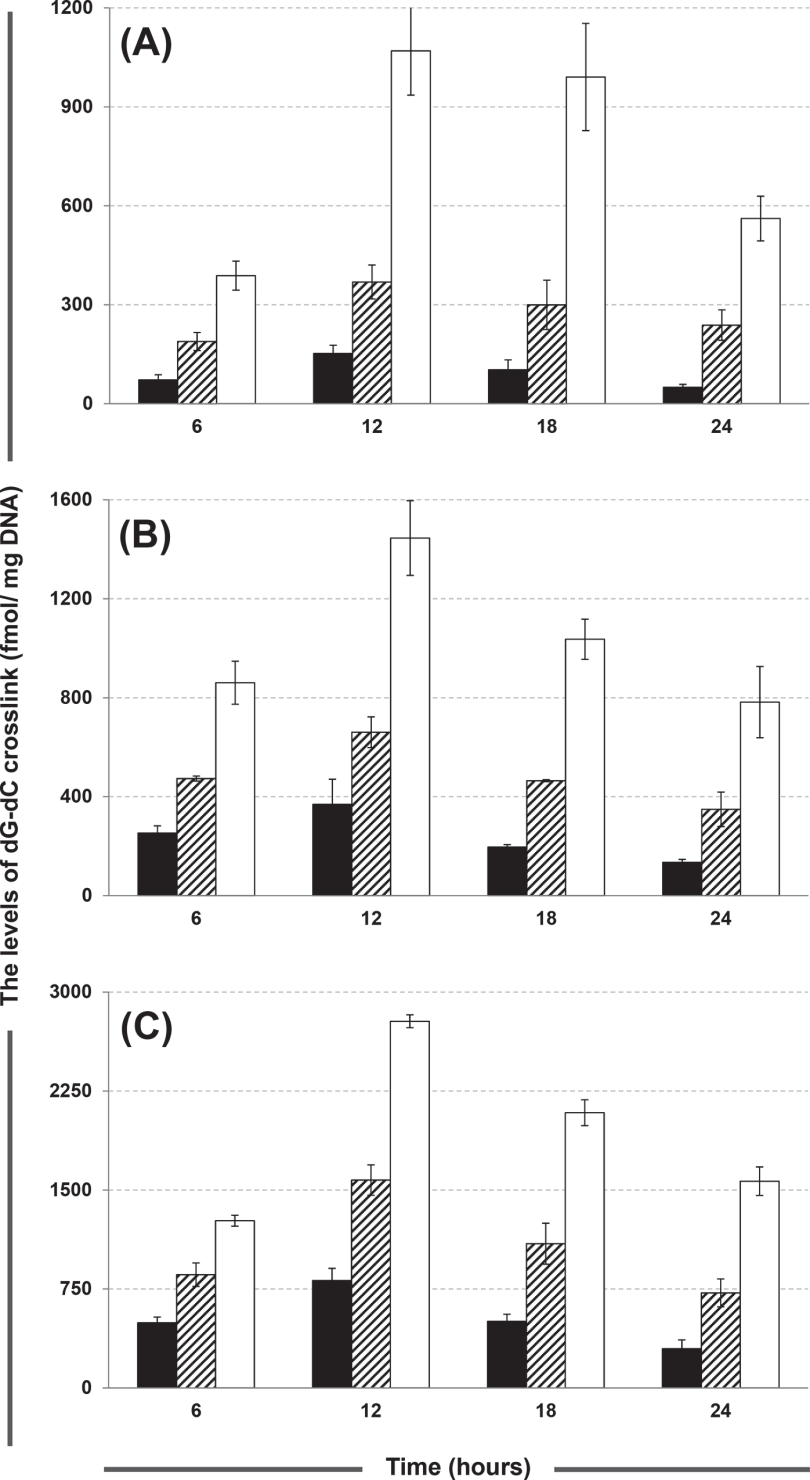
Comparison of the dG-dC crosslinking levels (fmol/mg DNA) in SF-126 (open bars), SF-767 (striped bars) and SF-763 (solid bars) cells treated with ACNU at the concentrations of 0.2 (A), 0.6 (B) and 1 (C) mM.

The levels of dG-dC crosslinks were determined in human glioma SF-767, SF-763 and SF-126 cells with ACNU exposure at doses of 0.2, 0.6 and 1 mM for 24 h. Fig. 4 displays the comparison of the levels of dG-dC crosslink in SF-763, SF-767 and SF-126 cells, and the corresponding values of the amounts of dG-dC crosslink at each time point are summarized in S3 Table. A dose-dependent formation of dG-dC crosslinks was observed when the concentration of ACNU increased from 0.2 to 1 mM. For the three types of cells, the maximal crosslinking levels were all observed at 12 h with the values of 153, 369 and 814 for SF-763 cells, 369, 661 and 1576 for SF-767 cells, and 1070, 1446 and 2779 for SF-126 cells, treated with 0.2, 0.6 and 1 mM ACNU, respectively. The levels of dG-dC crosslinks gradually decreased after 12 h; for some samples, even lower levels were observed at 24 h than at 6 h. The decrease of the crosslinking levels is mainly attributed to the dilution of the cells containing dG-dC crosslinks by cell proliferation during the later period of the treatment when ACNU was almost exhausted after 12 h. Moreover, in addition to the AGT-mediated repair mechanism, there are other pathways that can repair crosslinked base pair, such as the base excision repair (BER) pathway, in which the BER proteins are capable of recognizing and removing the crosslinked base pairs [41]. Similar decreasing trends have also been observed in previous studies. Ueda-Kawamitsu et al. [42] determined the BCNU-induced DNA ICLs in L1210 leukemia cells using a fluorescence assay, and demonstrated that the crosslinking level decreased after the maximum value observed at 6 h. Penketh et al. [28] also observed a decreasing trend following the maximal crosslinking levels in T7 DNA treated with BCNU, CCNU and MeCCNU using a fluorescent assay. However, in comparing our results with a study performed in our lab that treated calf thymus DNA with ACNU, no decreasing trend in the dG-dC crosslinking level was observed over 24 h (data not presented). This suggested that the dG-dC crosslinks are stable and will not decompose in the reaction mixture once formed. Therefore, it was postulated that the decrease in the crosslinking levels in the three types of human glioma cells could be attributed to the cell proliferation after the exhaustion of ACNU and probably non-AGT mediated repair.

To investigate the influence of AGT expression level on the formation of dG-dC crosslinking level, human glioma cells SF-126 (low AGT expression), SF-763 (medium AGT expression) and SF-767 (high AGT expression) were treated with ACNU. Control western blot analysis was performed to confirm the differences in AGT levels between the cell lines. The results indicated that SF-763 and SF-767 contained 2.1 and 1.6 times (P<0.01) the amount of AGT in SF-126, respectively (see S4 Fig.). As shown in Fig. 4, a significant difference in the crosslinking levels was observed between the three types of cells, which have different AGT contents, at each time point. In the repair-deficient SF-126 cells treated with 0.2 mM ACNU, the levels of dG-dC crosslink ranged from 388 to 1070 fmol/mg DNA and was 2.1 to 3.3 times (P<0.01) higher than those in SF-767 cells (189 to 369 fmol/mg DNA) and 5.4 to 11.2 times (P<0.01) higher than those in SF-763 cells (50 to 153 fmol/mg DNA). For the groups treated with 0.6 and 1 mM ACNU, the levels of dG-dC crosslink in SF-126 cells were 1.5 to 2.2 times (P<0.05) higher than those in SF-767 cells and 2.6 to 5.8 times (P<0.01) higher than those in SF-763 cells. This result was consistent with previous studies [43,44], which presumed that the levels of dG-dC crosslink in glioblastoma cells depended not only on the dose of alkylating agent, but also on AGT expression. Erickson et al. [45] measured the levels of DNA ICL and DNA-protein crosslinks in CENU-treated human cell strains from malignant tumors using alkaline elution assays. They found that the strains deficient in AGT produced consistently higher levels of DNA ICL than the AGT-proficient strains. Bodell et al. [12] compared the amounts of CENU-induced dG-dC crosslink in three rat glioma cell lines, 9L, 9L-2 and BTRC-19. The results indicated that the dG-dC crosslink amounts in 9L-2 and BTRC-19 cells were reduced by 65% and 79%, respectively, when compared to that in 9L cells. This reduction was considered to be associated with the increased AGT activity in 9L-2 and BTRC-19 cells. All of these studies revealed that the formation of DNA ICL induced by CENUs was closely related to the AGT levels in different cell lines. Therefore, the level of dG-dC crosslink, which is influenced by the expression levels of the AGT protein in tumor cells, can be employed as a biomarker for monitoring the drug resistance of CENUs in the clinical treatment of cancer.

**Fig 5:**
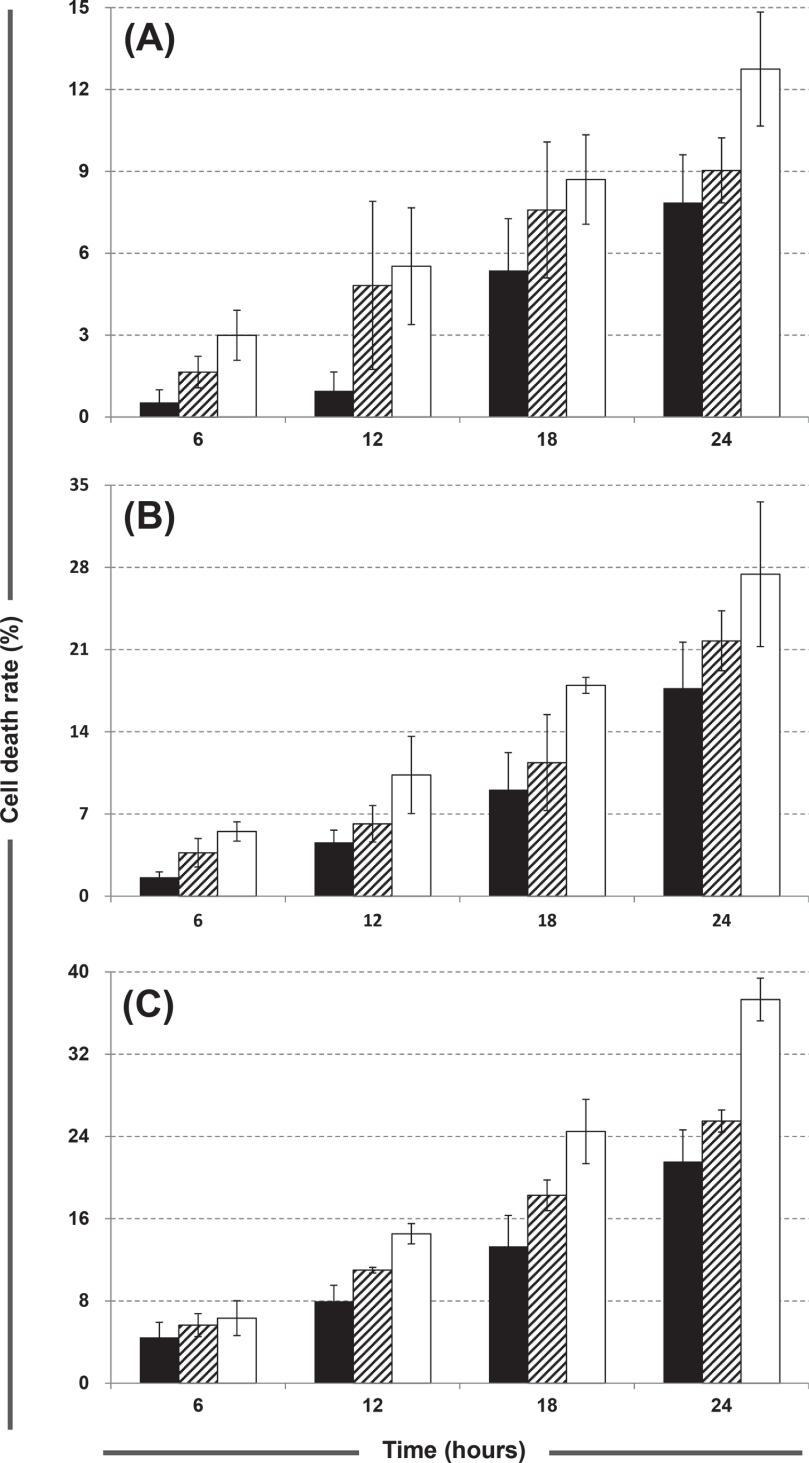
Cell death rates for SF-126 (open bars), SF-767 (striped bars) and SF-763 (solid bars) cells exposed to ACNU at the concentrations of 0.2 (A), 0.6 (B) and 1 (C) mM.

A trypan blue exclusion assay was performed to determine the cytotoxicity. As illustrated in Fig. 5 (the data are listed in S4 Table), the death rates of the SF-126 groups treated with ACNU at all drug concentrations are significantly higher than those of the SF-767 and SF-763 groups at all time points. This result agrees with the observation that SF-126 has higher crosslinking levels than SF-767 and SF-763. Therefore, it was inferred that the low levels of dG-dC crosslink formation in AGT-proficient cells is a crucial factor for the low cytotoxicity, which leads to drug resistance of CENUs. However, Fig. 4 demonstrates that the levels of dG-dC crosslink for all groups reach a maximum at 12 h, while the cell death rates continue to increase with exposure time. The primary cause of the discrepancy is that serum was added to the culture media to keep the cells growing during the treatment to ensure that plenty of cells could be harvested for DNA extraction; while in the cytotoxicity assay, the cells were incubated in the culture media containing ACNU without serum addition. Therefore, it is plausible that the decrease in the crosslinking levels after 12 h can be attributed to the proliferation of cells, as mentioned above.

The results arising form the comparative experiments obtained by HPLC-ESI-MS/MS and cytotoxicity assay provide direct evidences for the correlation within AGT level, dG-dC crosslinking level and cytotoxicity. CENUs exhibit higher cytotoxicity by inducing higher levels of dG-dC crosslink in AGT-deficient cells compared with AGT-proficient cells, owing to the repair of O^6^-alkylated guanine by abundant AGT before the formation of crosslink. The results not only definitely explain the resistance of AGT-proficient cells to CENUs, but also present a challenge that novel O-6 modifying anticancer agents with the ability of depleting AGT are required for overcoming the drug resistance in clinical application. Recently, base upon the structure of laromustin, a dual function alkylators with a moiety of *N*-nitrosourea was synthesized and observed superior cytotoxicity against repair proficient tumor cells [46]. The compound contains not only a chloroethyl group for crosslinking DNA as CENUs, but also a methyl group with the ability of efficiently depleting AGT in high AGT expressing DU145 human prostate carcinoma cells. O^6^-benzylguanine (O^6^-BG) is an effective AGT inhibitor and was clinically used to sensitize tumors to CENUs. Previous studies from our laboratory demonstrated that higher crosslinking level and higher cytotoxicity were observed in SF-767 cells pretreated with O^6^-BG compared to those without O^6^-BG pretreatment [47]. This corroborates the results obtained in the present work that high levels of dG-dC crosslink leading to high cytotoxicity can be achieved by effectively ablating AGT expression in cancer cells.

## Conclusions

In this study, quantitative analyses were performed to determine the dG-dC crosslinks induced by CENUs in mouse L1210 leukemia cells and human SF-126, SF-763 and SF-767 glioma cells using HPLC-ESI-MS/MS. The results indicated that the supposed quantitative method is feasible for determining dG-dC crosslinks in various types of cells with satisfying sensitivity and specificity. In comparing the four types of CENUs, we found that ACNU has higher crosslinking potency than BCNU, CCNU and FTMS. This was consistent with the results of a previous cohort study, which reported that ACNU resulted in a higher survival gain for high-grade glioma patients compared to the other three CENUs. In SF-126, SF-763 and SF-767 cells treated with ACNU, the crosslinking levels showed dose-dependence, and reached the maximum at 12 h in all groups. A common decreasing trend was observed after the maximal crosslinking levels achieved, which was attributed mainly to the cell proliferation and probably to the non-AGT mediated repair. Significant differences were observed between the crosslinking levels in the three cell lines. The highest crosslinking levels were observed in the AGT-deficient SF-126 cells, while the lowest levels were observed in the AGT-proficient SF-763 cells. This is consistent with the results of the corresponding cytotoxicity assay, which demonstrated that SF-126 has the highest cell death rates, followed by SF-767 and SF-763. The results presented in this study indicate that the levels of dG-dC crosslink are not only associated with anticancer efficiency, but also reflect AGT-mediated drug resistance. This work is expected to contribute to the further understanding of drug resistance to CENU-based chemotherapies and will assist in the development of new bifunctional alkylating agents with high anticancer efficiencies.

## Supporting Information

S1 Fig Positive ESI product ion spectra of the [M+H]+ ions of dG-dC (A) and ^15^N_3_-dG-dC (B). (TIF)

S2 Fig SRM chromatograms for the dG-dC standard and ^15^N_3_-dG-dC internal standard. (TIF)

S3 Fig Calibration curve for dG-dC crosslinks constructed by plotting SRM peak area ratios versus the concentration ratio between dG-dC and ^15^N_3_-dG-dC. (TIF)

S4 Fig Western blot confirmation of the difference in AGT expression levels between SF-763, SF-767 and SF-126 cells. GAPDH was used as a loading control. Graphs at bottom present the Western blot analysis quantitative data (n = 3). Data is expressed as mean ± SD.
(TIF)

S1 Table CENU chemotherapies used in the clinical treatment of cancer and in preclinical study. (DOC)

S2 Table The determined levels of dG-dC crosslinks in L1210 cells treated with ACNU, BCNU, CCNU and FTMS at various drug concentrations. (DOC)

S3 Table The determined levels of dG-dC crosslinks in SF-763, SF-767 and SF-126 cells exposed to ACNU at various drug concentrations. (DOC)

S4 Table The cell death rates of SF-763, SF-767 and SF-126 cells exposed to ACNU at various drug concentrations. (DOC)
