# Supplementary figures and images for: Influence of the Expression Level of O6-Alkylguanine-DNA Alkyltransferase on the Formation of DNA Interstrand Crosslinks Induced by Chloroethylnitrosoureas in Cells: A Quantitation Using High-Performance Liquid Chromatography-Mass Spectrometry
Source: PLoS One. 2015 Mar 23;10(3):e0121225. doi: 10.1371/journal.pone.0121225 (PMC4370500; doi:10.1371/journal.pone.0121225)

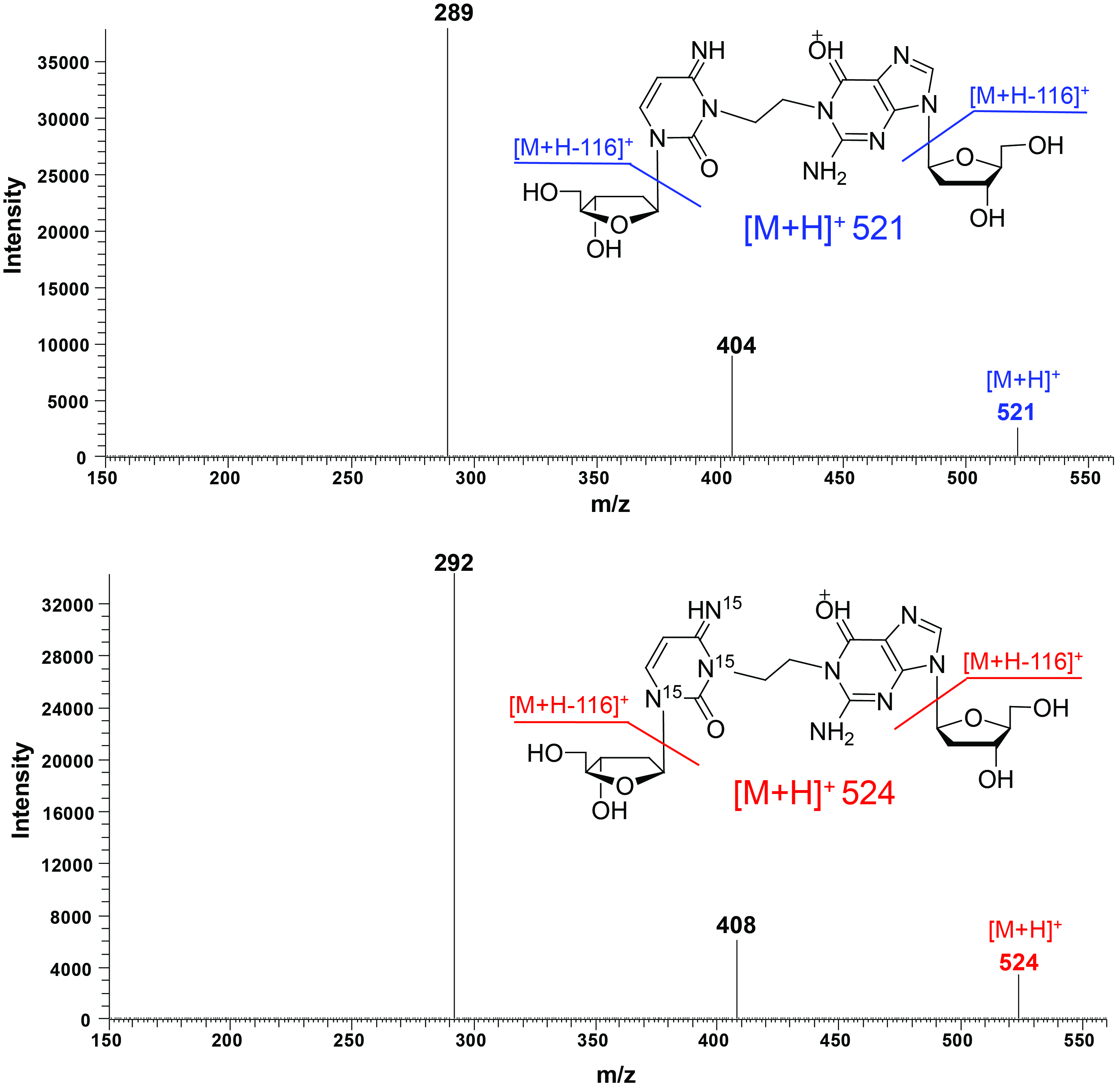

Supplement: S1 Fig — (TIF) [file pone.0121225.s001.tif]

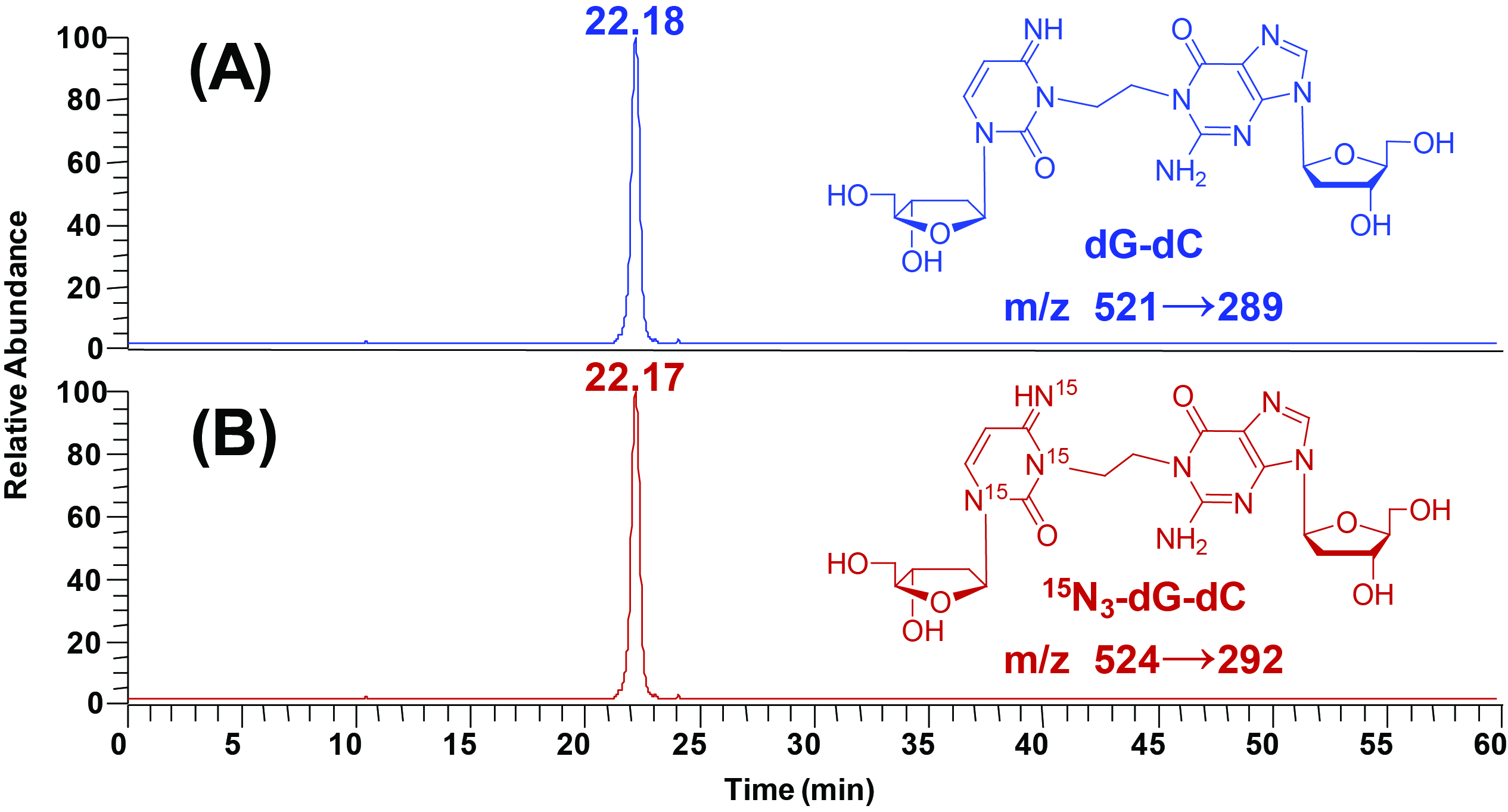

Supplement: S2 Fig — (TIF) [file pone.0121225.s002.tif]

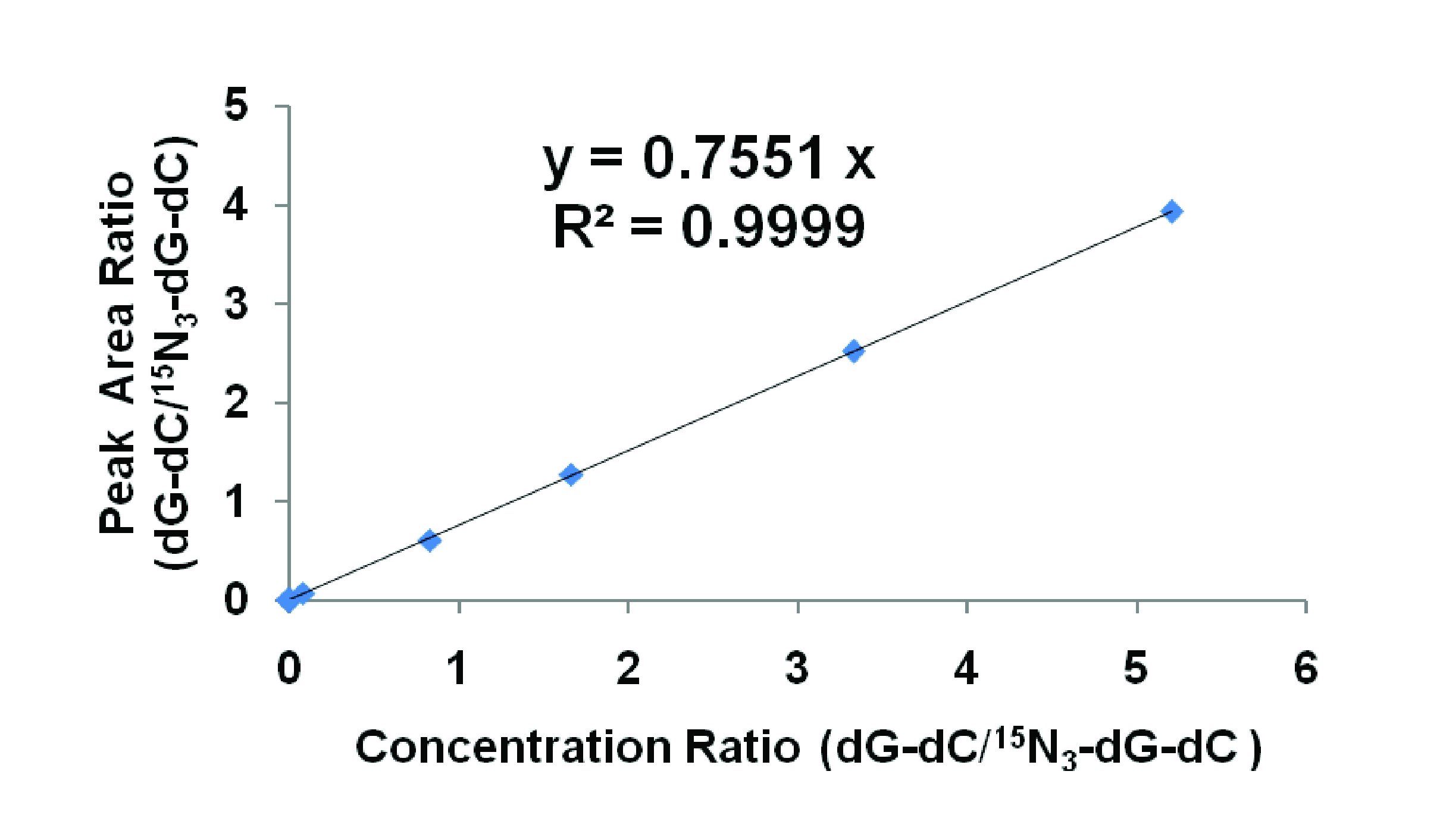

Supplement: S3 Fig — (TIF) [file pone.0121225.s003.tif]

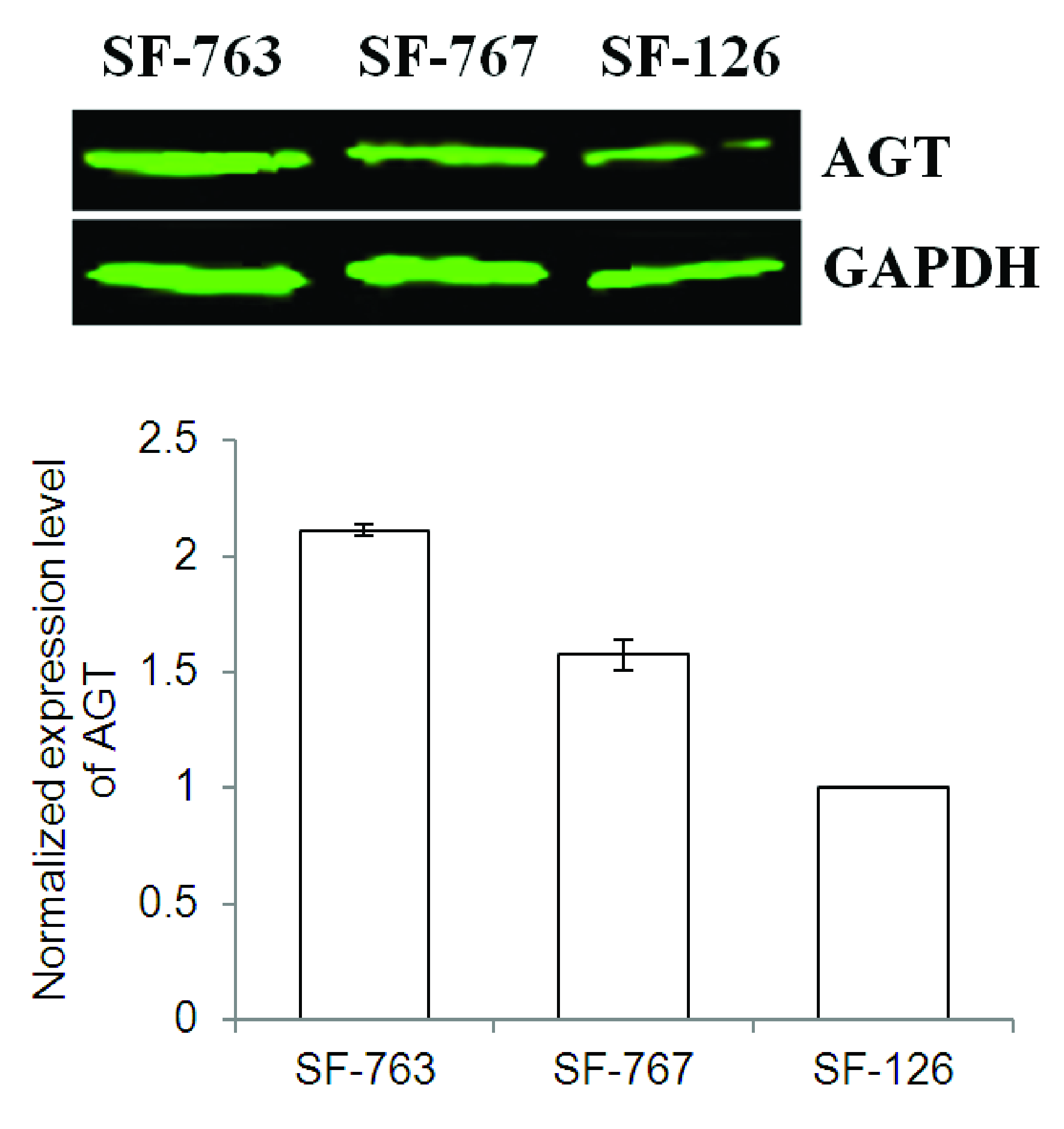

Supplement: S4 Fig — GAPDH was used as a loading control. Graphs at bottom present the Western blot analysis quantitative data (n = 3). Data is expressed as mean ± SD. (TIF) [file pone.0121225.s004.tif]
