# Supplementary material for: Influence of the Expression Level of O6-Alkylguanine-DNA Alkyltransferase on the Formation of DNA Interstrand Crosslinks Induced by Chloroethylnitrosoureas in Cells: A Quantitation Using High-Performance Liquid Chromatography-Mass Spectrometry
Source: PLoS One. 2015 Mar 23;10(3):e0121225. doi: 10.1371/journal.pone.0121225 (PMC4370500; doi:10.1371/journal.pone.0121225)
Supplement: S1 Table — (DOC) [file pone.0121225.s005.doc]

**S1 Table. CENU chemotherapies used in the clinical treatment of cancer and in preclinical study.**

| **Proprietary name and abbreviation** | **Molecular formula** | **Chemical structures** | **Indications** |
| --- | --- | --- | --- |
| **Nimustine**  **(ACNU)** | C_9_H_13_ClN_6_O_2_ |  | Brain tumors,  small cell lung cancer and Hodgkin's disease |
| **Carmustine**  **(BCNU)** | C_5_H_9_Cl_2_N_3_O_2_ |  | Brain tumors and Hodgkin's disease |
| **Lomustine**  **(CCNU)** | C_9_H_16_ClN_3_O_2_ |  | Brain tumors |
| **Semustine**  **(MeCCNU)** | C_10_H_18_ClN_3_O_2_ |  | Brain tumors |
| **Fotemustine**  **(FTMS)** | C_9_H_19_ClN_3_O_5_P |  | Brain tumors  and melanoma |
| **SarCNU** | C_6_H_11_ClN_4_O_3_ |  | Malignant glioma |
| **Ranimustine**  **(MCNU)** | C_10_H_13_ClN_3_C_7_ |  | Brain tumors, myeloma, leukemia,  and malignant lymphoma, |
| **Tauromustine**  **(TCNU)** | C_7_H_15_ClN_4_O_4_S |  | Myeloma, malignant glioma and lymphoma |
| **Chlorozotocin**  **(DCNU)** | C_9_H_16_ClN_3_O_7_ |  | Pancreatic tumors |
