# Supplementary material for: Influence of the Expression Level of O6-Alkylguanine-DNA Alkyltransferase on the Formation of DNA Interstrand Crosslinks Induced by Chloroethylnitrosoureas in Cells: A Quantitation Using High-Performance Liquid Chromatography-Mass Spectrometry
Source: PLoS One. 2015 Mar 23;10(3):e0121225. doi: 10.1371/journal.pone.0121225 (PMC4370500; doi:10.1371/journal.pone.0121225)
Supplement: S2 Table — (DOC) [file pone.0121225.s006.doc]

**S2 Table. The determined levels of dG-dC crosslinks in L1210 cells treated with ACNU, BCNU, CCNU and FTMS at various drug concentrations** (fmol/mg DNA)

| CENUs | Reaction time (hour) | Drug concentrations (mM) | | | | | |
| --- | --- | --- | --- | --- | --- | --- | --- |
|  |  | 0.025 | 0.05 | 0.1 | 0.2 | | 0.4 |
|  |  |  |  |  |  |  | |
| ACNU | 3 | 45±15 | 131±12 | 146±34 | 366±47 | 693±35 | |
|  | 6 | 109±40 | 190±42 | 462±76 | 545±21 | 976±51 | |
|  | 9 | 110±23 | 330±22 | 637±81 | 885±50 | 1271±115 | |
|  | 12 | 150±30 | 589±66 | 848±37 | 1124±122 | 1832±50 | |
|  |  |  |  |  |  |  | |
| BCNU | 3 | 22±2 | 51±21 | 83±44 | 183±29 | 282±75 | |
|  | 6 | 46±3 | 160±15 | 279±37 | 319±83 | 704±34 | |
|  | 9 | 69±18 | 186±37 | 465±79 | 802±42 | 985±18 | |
|  | 12 | 147±30 | 386±35 | 504±16 | 981±37 | 1243±55 | |
|  |  |  |  |  |  |  | |
| CCNU | 3 | 23±4 | 31±2 | 42±2 | 153±25 | 339±54 | |
|  | 6 | 48±5 | 120±9 | 118±60 | 226±103 | 459±53 | |
|  | 9 | 67±2 | 147±15 | 345±31 | 419±46 | 527±78 | |
|  | 12 | 121±2 | 211±73 | 427±32 | 487±61 | 783±61 | |
|  |  |  |  |  |  |  | |
| FTMS | 3 | 26±15 | 52±12 | 84±55 | 180±54 | 400±38 | |
|  | 6 | 50±0 | 178±38 | 355±11 | 475±84 | 872±67 | |
|  | 9 | 110±6 | 318±8 | 496±45 | 830±25 | 1187±107 | |
|  | 12 | 182±31 | 540±20 | 681±5 | 930±36 | 1387±81 | |
