# Supplementary material for: Influence of the Expression Level of O6-Alkylguanine-DNA Alkyltransferase on the Formation of DNA Interstrand Crosslinks Induced by Chloroethylnitrosoureas in Cells: A Quantitation Using High-Performance Liquid Chromatography-Mass Spectrometry
Source: PLoS One. 2015 Mar 23;10(3):e0121225. doi: 10.1371/journal.pone.0121225 (PMC4370500; doi:10.1371/journal.pone.0121225)
Supplement: S3 Table — (DOC) [file pone.0121225.s007.doc]

**S3 Table. The determined levels of dG-dC crosslinks in SF-763, SF-767 and SF-126 cells exposed to ACNU at various drug concentrations**

| Cell samples | Reaction time (hour) | Concentrations of ACNU (mM) | | |
| --- | --- | --- | --- | --- |
|  |  | 0.2 | 0.6 | 1 |
|  |  |  |  |  |
| SF-763 | 6 | 73±15 | 253±29 | 494±42 |
|  | 12 | 153±25 | 369±101 | 814±93 |
|  | 18 | 103±30 | 196±10 | 506±53 |
|  | 24 | 50±9 | 135±12 | 297±67 |
|  |  |  |  |  |
| SF-767 | 6 | 189±27 | 473±10 | 858±89 |
|  | 12 | 369±52 | 661±62 | 1576±115 |
|  | 18 | 300±75 | 465±4 | 1094±155 |
|  | 24 | 238±46 | 349±69 | 721±105 |
|  |  |  |  |  |
| SF-126 | 6 | 388±44 | 861±87 | 1267±42 |
|  | 12 | 1070±134 | 1446±151 | 2779±49 |
|  | 18 | 991±162 | 1037±81 | 2086±98 |
|  | 24 | 562±68 | 783±144 | 1567±108 |
