# Supplementary material for: Influence of the Expression Level of O6-Alkylguanine-DNA Alkyltransferase on the Formation of DNA Interstrand Crosslinks Induced by Chloroethylnitrosoureas in Cells: A Quantitation Using High-Performance Liquid Chromatography-Mass Spectrometry
Source: PLoS One. 2015 Mar 23;10(3):e0121225. doi: 10.1371/journal.pone.0121225 (PMC4370500; doi:10.1371/journal.pone.0121225)
Supplement: S4 Table — (DOC) [file pone.0121225.s008.doc]

**S4 Table. The cell death rates of SF-763, SF-767 and SF-126 cells exposed to ACNU at various drug concentrations**

| Cell samples | Reaction time (hour) | Concentrations of ACNU (mM) | | |
| --- | --- | --- | --- | --- |
|  |  | 0.2 | 0.6 | 1 |
|  |  |  |  |  |
| SF-763 | 6 | 0.5±0.5 | 1.6±0.5 | 4.5±1.5 |
|  | 12 | 1.0±0.7 | 4.6±1.0 | 8.0±1.5 |
|  | 18 | 5.4±1.9 | 9.1±3.2 | 13.3±3.0 |
|  | 24 | 7.9±1.8 | 17.7±3.9 | 21.6±3.1 |
|  |  |  |  |  |
| SF-767 | 6 | 1.6±0.6 | 3.7±1.2 | 5.6±1.1 |
|  | 12 | 4.8±3.1 | 6.2±1.6 | 11.0±0.3 |
|  | 18 | 7.6±2.5 | 11.4±4.1 | 18.3±1.5 |
|  | 24 | 9.0±1.2 | 21.8±2.5 | 25.5±1.1 |
|  |  |  |  |  |
| SF-126 | 6 | 3.0±0.9 | 5.5±0.8 | 6.3±1.7 |
|  | 12 | 5.5±2.1 | 10.3±3.3 | 14.5±1.0 |
|  | 18 | 8.7±1.6 | 18.0±0.7 | 24.5±3.1 |
|  | 24 | 12.7±2.1 | 27.4±6.2 | 37.3±2.1 |
